# See, Like, Share, Remember: Adolescents’ Responses to Unhealthy-, Healthy- and Non-Food Advertising in Social Media

**DOI:** 10.3390/ijerph17072181

**Published:** 2020-03-25

**Authors:** Gráinne Murphy, Ciara Corcoran, Mimi Tatlow-Golden, Emma Boyland, Brendan Rooney

**Affiliations:** 1 Media and Entertainment Lab, School of Psychology, University College Dublin, Belfield, 4 Dublin, Ireland; 2 Faculty of Wellbeing, Education and Language Studies, The Open University, Milton Keynes MK7 6AA, UK; 3 Institute of Psychology, Health and Society, University of Liverpool, Liverpool L69 7ZA, UK

**Keywords:** marketing, advertising, social media, adolescent, food, recall, attention, peers, sharing, obesity

## Abstract

Media-saturated digital environments seek to influence social media users’ behaviour, including through marketing. The World Health Organization has identified food marketing, including advertising for unhealthy items, as detrimental to health, and in many countries, regulation restricts such marketing and advertising to younger children. Yet regulation rarely addresses adolescents and few studies have examined their responses to social media advertising. In two studies, we examined adolescents’ attention, memory and social responses to advertising posts, including interactions between product types and source of posts. We hypothesized adolescents would respond more positively to unhealthy food advertising compared to healthy food or non-food advertising, and more positively to ads shared by peers or celebrities than to ads shared by a brand. Outcomes measured were (1a) *social responses* (likelihood to ‘share’, attitude to peer); (1b) *brand memory* (recall, recognition) and (2) *attention* (eye-tracking fixation duration and count). Participants were 151 adolescent social media users (Study 1: *n* = 72; 13–14 years; M = 13.56 years, SD = 0.5; Study 2: *n* = 79, 13–17 years, *M* = 15.37 years, *SD* = 1.351). They viewed 36 fictitious Facebook profile feeds created to show age-typical content. In a 3 × 3 factorial design, each contained an advertising post that varied by content (healthy/unhealthy/non-food) and source (peer/celebrity/company). Generalised linear mixed models showed that advertisements for unhealthy food evoked significantly more positive responses, compared to non-food and healthy food, on 5 of 6 measures: adolescents were more likely to wish to ‘share’ unhealthy posts; rated peers more positively when they had unhealthy posts in their feeds; recalled and recognised a greater number of unhealthy food brands; and viewed unhealthy advertising posts for longer. Interactions with sources (peers, celebrities and companies) were more complex but also favoured unhealthy food advertising. Implications are that regulation of unhealthy food advertising should address adolescents and digital media.

## 1. Introduction

The prevalence of overweight and obesity in young people is rising globally with consequences for long-term health [1,2]. There is strong evidence that marketing, including advertising, for unhealthy food (high in saturated fat, salt or sugar: HFSS) contributes to overweight and obesity [2,3,4], and a consensus is increasingly developing that the persuasive actions marketers engage in, to influence children’s (including adolescents’) behaviour, infringes children’s rights, including rights to health and not to be exploited [5,6]. However, much of the existing evidence for young people’s interactions with marketing and its effects has been generated for television and for younger children rather than adolescents [3]. Yet young people spend increasing amounts of time engaged in online activities [7,8,9].

Advertisers have extensive digital media presence including on social and media-sharing platforms where they promote products and brands as exciting and interactive [6,10,11]. In digital media (as in traditional media), most food and beverage advertising is for unhealthy items: reports indicate 65%–80% of food advertising online is for HFSS products or brands associated with these foods [12,13,14]. Furthermore, as food and beverage companies have extensive followings online, including among teens, their activities reach large audiences [15]; the food brands with the greatest potential reach amongst teens are almost all brands with many or mostly unhealthy products in their portfolios [16]. Adolescents are at risk of exposure to unhealthy food advertising because of their very high levels of Internet and social media usage. Diary, screen-recording and avatar studies indicate high levels of exposure [12,14,17]. However, evidence for how young people engage with and respond to food advertising in digital media remains limited [4,6].

Although adolescents understand the persuasive intent of advertising, they are hypothesized to lack the motivation and ability to defend against its effects [6,18,19]. Alcohol and tobacco advertising research suggests that moderation of advertising influence is dependent on viewers’ self-control [20], a quality often still developing in adolescence. Research also points to hypersensitivity to reward in the adolescent years [21]. Furthermore, specific features of digital media advertising may reduce cognitive defences to effects of marketing [22]. Brands on social media regularly create interactive content not present in traditional media [6,11] which is highly integrated and often difficult to distinguish from non-marketing content [23]. Online marketing also engages with users’ social networks, inserting themselves into adolescents’ social lives by presenting brands as ‘liked’ by friends and encouraging users to interact with brands as if they were individuals [24,25]. Thus, despite being advertising-literate, adolescents are likely to be vulnerable to food advertising.

### 1.1. Adolescents and Peers Online

Adolescents are particularly susceptible to social effects as they are motivated to interact with their peers [26] and, in social media, to connect with and view friends’ profiles [8]. Sharing social media content with friends serves a number of psychological incentives including self-expression and connecting with others [27]. Adolescents give careful consideration to the image they present online, conveying a socially acceptable self-image to others by sharing content popular with friends [28]. They place a great importance on peer norms and acceptance [29], identifying with their friends and generally with those of the same gender [30]. As social media sites allow users to connect with friends extensively [31], they are a powerful means for transmitting norms, ideas and behaviours.

The normative model of eating indicates that eating is directed by situational norms, the eating behaviours of those present, and their social approval [32]. Compared to preadolescents, teen peers exert more influence on food choice [33,34]: adolescents describe eating more unhealthy foods at school and with their friends than at home [35] and exchanges with peers stimulate unhealthy eating behaviours [36,37]: teens attempt to manage peers’ impressions of them through altering eating habits in order to meet what they perceive as the social norm [38,39]. Presenting or ‘sharing’ pictures of food is a popular activity in social media [40]. Peers are often thought to be more trustworthy than brands, and effects of online advertising are reported to be amplified when this is endorsed by a peer [6,41].

### 1.2. Celebrities

Social media allows users to interact not only with peers but view content posted by celebrities, who have role model status for young people [42]. Social media users can gain the illusion of a personal connection with celebrities, following updates in a similar way that they do from friends and family and with whom they may develop ‘parasocial’ relationships: for example, a study of fans’ interaction with the reality television personality Kim Kardashian’s online persona found they felt they were in a reciprocal, parasocial friendship [43,44]. Sports stars, music celebrities and online influencers regularly promote unhealthy food; up to one quarter of endorsements by music celebrities and athletes are promotions of HFSS foods and beverages [45,46], which can lead to increased consumption [47], often over healthier options [48].

### 1.3. Recall, Recognition and Attention

The food advertising hierarchy of effects framework [49] indicates that brand recall and recognition influence brand attitudes and eating behaviours, which lead to weight-related outcomes.

After advertising is viewed, it is retained in memory either explicitly (i.e., with conscious awareness) or implicitly [50,51]; and greater cognitive processing leads to easier recall [52].

As media use increases, however, and multiple-device viewing becomes the norm [7], it is reported that only 10% of all online advertisements are attended to [53] so it is important, rather than identifying the mere presence of marketing content in social media, to identify what is attended to. Eye-tracking is a widely used index of attentional selection [54] with longer and greater number of fixations associated with a more favourable opinion of the item [55]. Attention can lead to altered eating patterns in young people [56], and unhealthy food items attract greater interest than healthy and non-food items [57,58]. Social context is thought to play a significant role in ad recall, awareness and intent to purchase [41] but evidence is limited, particularly so for social context in the online space.

This study aimed to determine adolescents’ responses to healthy, unhealthy, and non-food advertising. The food advertising hierarchy of effects framework [49] synthesizes multiple theories and strands of empirical research to conclude that repeated exposure to advertising triggers recall and recognition, positive attitudes and normalization of promoted products, and subsequently, when exposed to relevant cues, intent to purchase or consume. Theories of social norms of eating can be nested within this model and these indicate that social groups establish norms for appropriate foods [59]. In social media, social norms of food are displayed, disseminated and reinforced, as young people do not just *see* food advertising but can also choose to *share* it with their ‘imagined audience’ of peers [27], and in turn can also *assess their peers* based on such content. Thus, the identity and self-presentation-based normative goals of the adolescent years [27] are interwoven in social media with food advertising.

Given the networked and fluid nature of social media, where – in contrast to broadcast media—advertising is presented to users not only from companies themselves but also via multiple other sources, including peers and celebrities who may be considered more trustworthy than brands, the study also examined effects of the *source* of advertising posts viewed.

The study investigated adolescents’ responses to advertisements for three *types of products* in social media: unhealthy food, healthier food, and non-food. It also measured effects of the *source* of these social media advertising posts. It is novel owing to its inclusion of healthy, unhealthy and non-food items, the social contexts of advertising received by adolescents in social media, and in combining objective measures of attention using eye-tracking technology not only with brand memory but also with self-report of social responses: we are unaware of any previous study to do this.

Assessing social responses, memory and attention, we hypothesized that participants will respond more positively to unhealthy food brands, compared to healthy food or non-food brands; and to advertising posts whose source appeared to be a celebrity or a fictional peer, rather than a brand or company.

## 2. Materials and Methods

### 2.1. Design

This mixed methods study involved two experimental studies (with outcome measures in three domains) designed to replicate a social media viewing experience. Both studies involved repeated measures true experiments with a 3 × 3 factorial design using a sequence of profile news feeds designed to mimic Facebook. In these, the *content* of target advertising posts varied systematically between healthy food, unhealthy food, and non-food; the *source* varied systematically between peer, celebrity, and company. Each combination of factors appeared four times (i.e., four trials) with a different brand each time (total 36 feeds). Adolescents’ responses to advertisements in social media were measured through three modalities: social responses, memory for brands, and attention to advertising posts. Dependent variables were

*Study 1a Social responses*
  (i) likelihood to ‘share’ advertising posts
  (ii) attitude to peer
*Study1b: Memory for brands*
  (iii) free brand recall
  (iv) prompted brand recognition
*Study 2: Attention to advertising*
  (v) mean fixation duration
  (vi) mean fixation count

### 2.2. Stimulus Material

**Figure 1:**
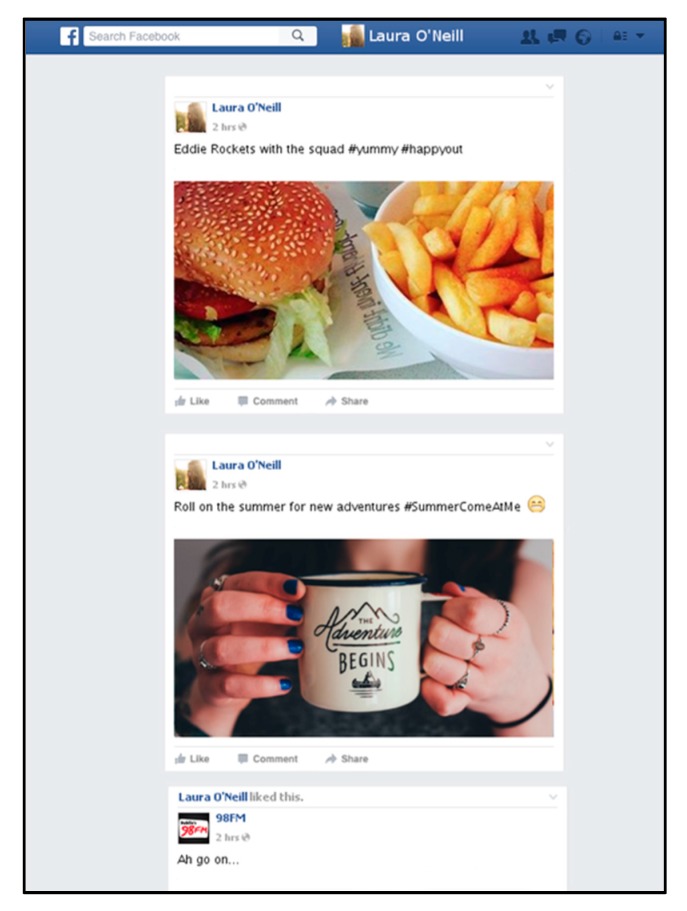
An example of a profile news feed image. The first post (burger and fries) is the target advertising post (in this case, unhealthy food advertisement, shared by a peer); the second and third are generic distractor posts.

*Facebook ‘News Feed’.* Facebook was the most widely used social media platform amongst adolescents in Ireland in the most comprehensive study available at the time of designing the materials [8]. Ecologically valid stimuli were created to resemble Facebook News Feeds of fictitious teen users (36 males and 36 females). To match Facebook’s design, each page contained a small profile picture and owner’s name (See Figure 1). Profile usernames were generated using common first names of the cohort identified in the Irish Central Statistics Office release for 2000 [60].

Each ‘profile view’ contained one advertising post (the target image), and two distractors. Each advertising post represented one content/source condition (e.g., unhealthy food ad, posted by a peer; or non-food ad, posted by a celebrity). For the two distractor posts, one was a full post with an image e.g., quotations, cartoons, status updates and images of people, animals and places. The other was text-only, shortened to give the impression that the feed continued below the screen.

In half the feeds, the advertising post appeared first. While the usernames and distractor images differed by gender, advertising images remained the same. To reduce potential confounds, the ‘like’, ‘share’ and ‘comment’ buttons contained no additional information, as ‘likes’ have been found to influence adolescents’ attitudes toward content.

### 2.3. Selection of Brands and Products for Advertising Posts

Food products were selected from local and international products widely available in local retail outlets and likely to be familiar to teenagers. World Health Organization 2015 Nutrient Profile Model guidelines for advertising to children [61] were applied to identify foods considered suitable to market to children such as snacks, breakfast cereals and fruit, and unsuitable items such as crisps (potato chips), chocolate and fast food. Non-food items were selected from those of interest to many young adolescents, such as technology, games, sports and cosmetics.

### 2.4. Selection of Sources for Advertising Posts

‘Peer’-originating advertising posts were created as if originating from other fictitious profile owners; ‘celebrity’ posts from celebrities representing music, sporting and movies likely to be popular with that age group; and ‘company’ posts from the brand or product of interest.

Two sets of social media feed images were developed so that male and female participants viewed gender-matched profile views. Celebrity posts were gender-matched to participants as research demonstrates favourability for celebrities of the same sex in adolescents [62,63]; however, the food brands viewed were identical. For example, Taylor Swift, a female singer, has promoted Subway sandwiches; Christiano Ronaldo, a male soccer player, has promoted the same product. In the non-food category advertising posts were for gender-normative items (e.g., clothing for females and computer games for males). Table 1 lists celebrities featured in the study and Table 2 lists all products shown in the advertising posts created for the study.

Finally, two young people aged 18 years (both female, accessed through personal contacts) reviewed all the profile views to consider authenticity for teens. Following their suggestions, some images were changed for more youth-oriented pictures; more hashtags, emojis, and exclamation marks were included in the text.

### 2.5. Ethics and Participant Recruitment

The study protocol was approved by the Ethics Committee of University College Dublin (Attitude: TGREC-PSY 2015-27; Memory: TGREC-PSY 2015-33; Eye-tracking: TGREC-PSY 2016-7).

All participants and their parents gave informed written consent before participation. The study information stated its aim to explore social media and advertising but did not indicate a focus on food advertising. Participants were debriefed after taking part.

Participants were recruited through secondary schools in Ireland (fee-paying and non-feeing paying, single-sex and co-educational schools), youth clubs and Facebook advertising. The youngest participants recruited were 13 years, the age at which social networks’ terms and conditions permit their use. Power analysis (G*Power) [64] demonstrated that to be sufficiently powered (1 − *β* = 0.8) to detect small effect sizes (f = 0.15), the current design required a total sample size of 39. Participants for Study 1 (*n* = 72) were aged 13–14 years; participants for Study 2 (*n* = 81) were aged 13–17 years.

## 3. Study 1: Social Responses and Memory

### 3.1. Participants and Procedure

Participating secondary schools in Ireland (*n* = 5) were in Dublin and Ennis, County Clare; 72 adolescents took part (45 females; M = 13.56 years, SD = 0.5). Of these, *n* = 60 completed media use questions and reported watching 1.29 (SD = 0.99) hours of television daily; 99% (*n* = 71) went online more than once a day.

Participants completed the experiment on a tablet or computer in their school classroom. Researchers invited them to view feeds of similar-aged Facebook users to explore teens’ social media use, asking them to scroll through these as they would during normal use, and they were presented with 36 profiles in randomised order. While viewing each profile feed participants answered questions:

- eliciting attitude to the fictional peer whose social media page they were viewing, and likelihood to share the posts they saw
- about their digital media use and knowledge
- to elicit brand recall and recognition

### 3.2. Study 1a: Social Responses

To assess intent to share advertising posts, for every post viewed (advertising posts and distractors) participants were asked “How likely would you be to share this post?”. The responses to advertising posts only were analysed. Responses were scored on a 5-point Likert scale (1 ‘very unlikely’ to 5 ‘very likely’). To assess attitudes to peers, participants were also asked, for each of the 36 fictitious peers “What kind of impression do you have of this person?” (5-point Likert scale, 1 ‘very negative’to 5 ‘very positive’).

#### 3.2.1. Analysis

Data were cleaned: 8 of the 72 participants appeared not understand the recall question (with responses such as ‘?’ or ‘X’) or chose not to answer it, and 4 did not attempt the recognition question; these were removed from the dataset. Trials were combined to produce average score within each of the 3 x 3 repeated categories. A generalised log linear mixed model was used to test the hypotheses and was separately generated for the two dependent variables of peer attitude and self-representation (likelihood of sharing). *Ad content* (Unhealthy food, Non-food, Healthy food) and *Source of ad* (Peer, Celebrity, Company) were the independent (predictor) variables. In both models, the product type, source type, gender, age, and internet use were fixed factors and participant ID as a random factor. Company-shared non-food brands were set as the baseline. This allowed for an exploration of how other forms of sharing compared to the condition that most closely resembled traditional advertising. Analyses explored these rates in conjunction with effects of the source of the advertising post.

#### 3.2.2. Attitude to Peer

**Figure 2:**
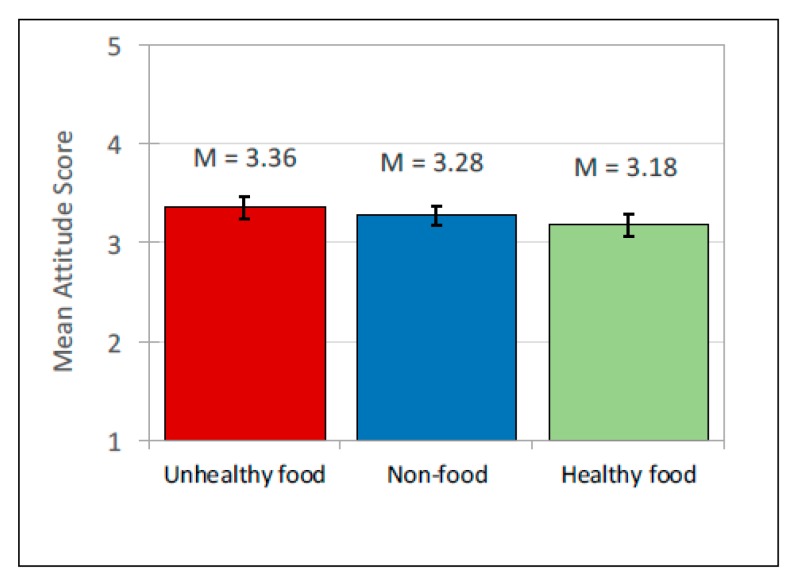
Attitude to peer: Mean scores.

**Figure 3:**
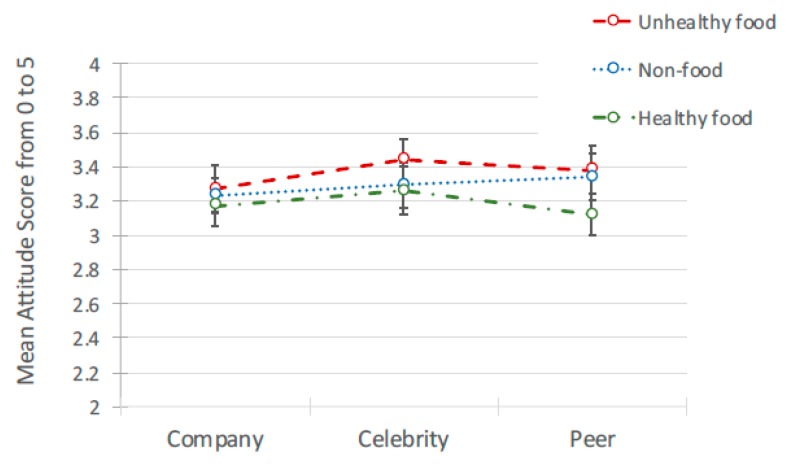
Interaction between Attitude to Peer and source of ad for 3 types of ad.

There was no significant interaction between ad content and the source of post. A significant main effect for *content* was observed, *F*(2, 636) = 14.28, *p* < 0.001. Participants rated peers with unhealthy food posts in their social media news feeds most positively and users with healthy food posts least positively (see Table 3 for means and Table 4 for pairwise comparisons; Figure 2 for means and Figure 3 for interactions). A significant effect of *source* of advertising post on attitude towards the user was also observed, *F*(2, 636) = 4.97, *p* < 0.01. Participants attitude to peers was significantly lower where social media profiles contained company-sponsored posts compared to peer or celebrity posts. Peer and celebrity posts did not differ significantly. There were no significant effects of participants’ age, gender and self-reported frequency of internet use.

#### 3.2.3. Likelihood to ‘Share’

**Figure 4:**
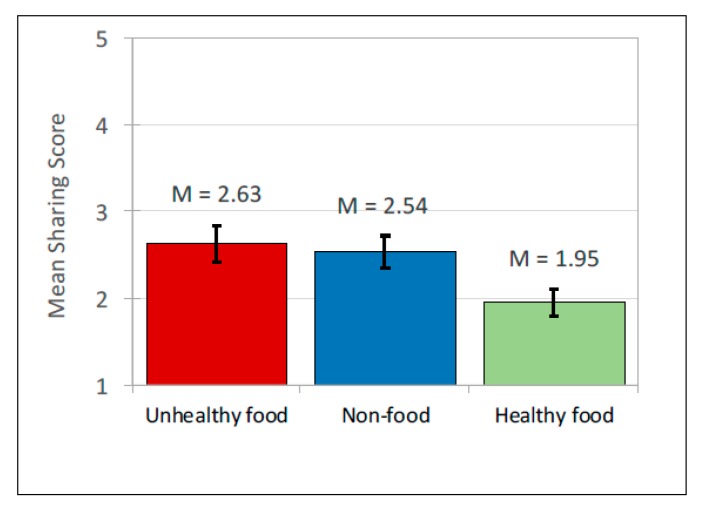
Likelihood to share: Mean scores.

**Figure 5:**
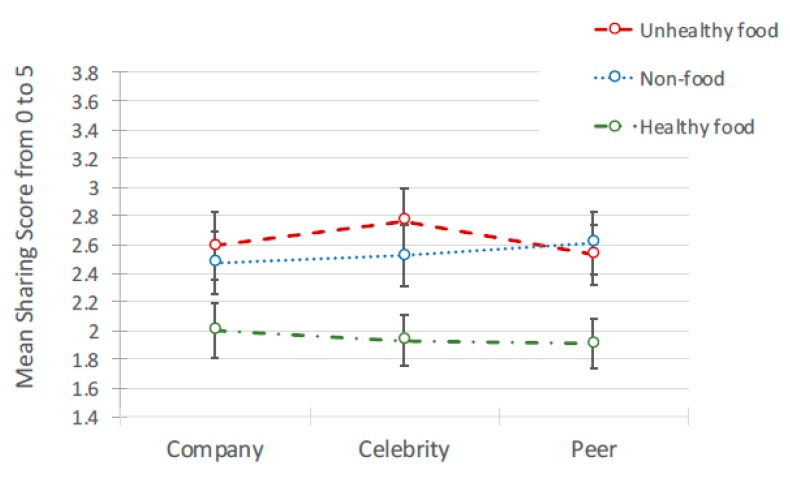
Interaction between Likelihood to Share and source of ad for 3 types of ad.

As with attitudes to peers, there was no significant interaction between ad content and the source of post affecting likelihood to share. A significant main effect of ad content was found, *F*(2, 636) = 101.27, *p* < 0.001, but no significant effect of source (see Table 3 for means and Table 4 for pairwise comparisons; Figure 4 for means and Figure 5 for interactions). Pairwise comparisons showed that participants reported they were significantly less likely to share healthy advertising posts than unhealthy and non-food posts, with unhealthy food posts marginally higher than non-food. Age, gender and frequency of internet use again revealed no significant effects.

### 3.3. Study 1b: Brand Memory

Study 1b sought to identify participants’ memory for brands they had viewed. At the end, after completing a short survey with closed and open questions about their internet use, participants were asked to

i list all brands they recalled having seen while viewing the Facebook feeds and
ii select the brands they recalled having seen in the profiles they had just viewed, from a list of 56 brands (the 36 target brands and 20 similar distractors not used in the study).

The dependent variables were therefore (i) free brand recall (ii) recognition.

#### 3.3.1. Data Analysis

Data were managed as described for Study 1a. For *Recall* (*n* = 64), participants’ accurate recall was *M* = 3.2 (*SD* = 1.9) of the 36 brands (of *M* = 4.14, *SD* = 2.19 brands they listed). For *Recognition* (*n* = 68), participants correctly recognised *M* = 15.84 (*SD* = 6.38) of the 36 brands (of *M =* 18.21, *SD* = 8.08 responses). (see Table 5 for means). Of the 36 brands shown, the mean *recall* rate for unhealthy brands (1.75) was nearly five times that for healthy brands (0.36). It was also greater for non-food brands (1.09). Similarly, for prompted *recognition*, the mean number of unhealthy brands recognised (7.53) was double that for healthy brands (3.87); non-food brands (4.44) were also recognised more than healthy brands. Analyses explored these rates in conjunction with effects of the source of the advertising post. The independent (predictor) variables were ad *content* (unhealthy food, healthy food, non-food) and *source* of post (peer, celebrity, company). A generalized mixed model was generated separately for recall and recognition. Both used product type, source type, gender, age, and internet use as fixed factors and participant ID as a random factor. Non-food brands shown in company-source posts were set as the baseline.

#### 3.3.2. Free Recall

**Figure 6:**
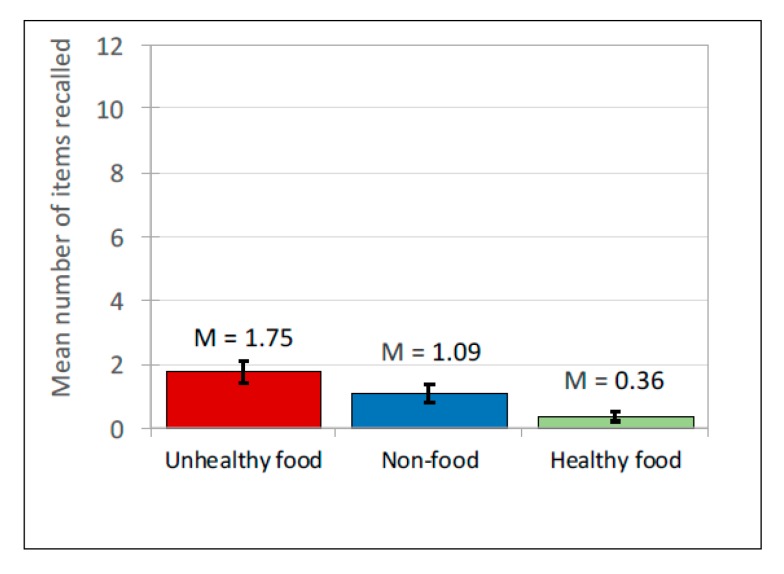
Free brand recall: Mean scores.

**Figure 7:**
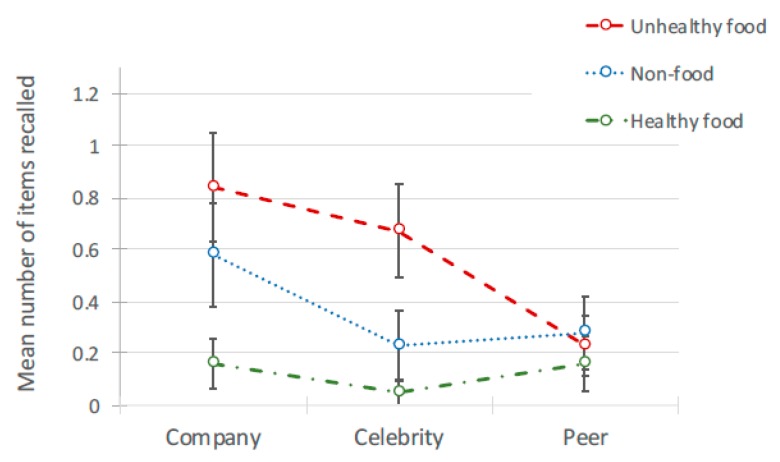
Interaction between brand recall and source of ad for 3 types of ad.

There was a significant main effect of ad content on free recall, *F*(2, 582) = 22.582, *p* < 0.001. Free recall was low overall but highest for unhealthy food and lowest for healthy food brands (see Table 5 and Figure 6). The model also demonstrated that ad *content* interacted with the *source* of the social media post, *F*(4, 582) = 3.724, *p* < 0.01 (see Table 6 for pairwise comparisons and Figure 7). The effects disappeared when looking just to peers, where there was no significant differences between the ad content conditions. For posts from celebrities and companies, unhealthy food posts were recalled more than healthy posts, with non-food posts recalled least.

**Figure 8:**
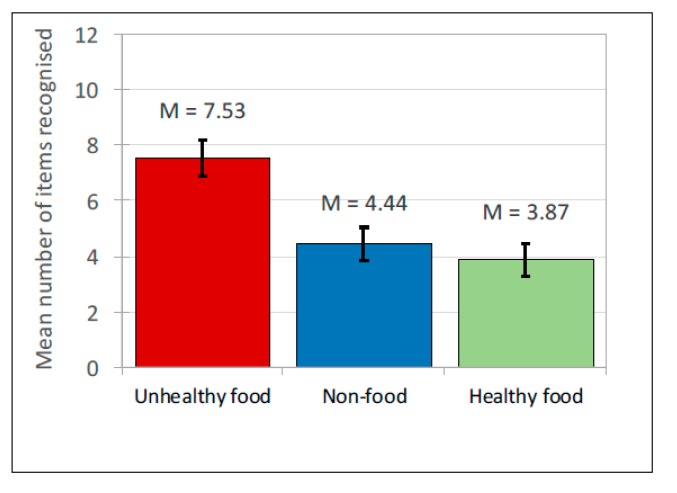
Prompted brand recognition: Mean scores.

**Figure 9:**
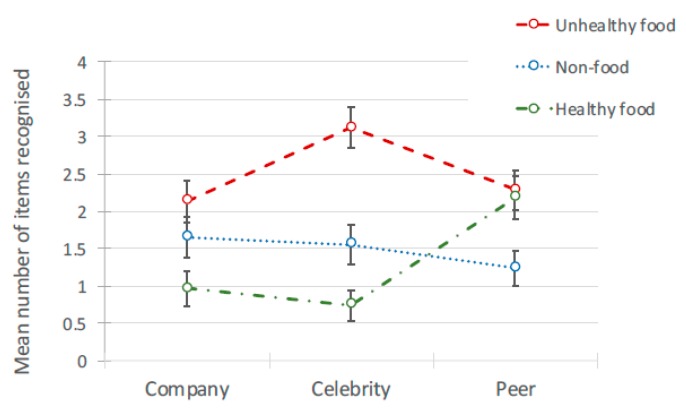
Interaction between prompted brand recognition and source of ad for 3 types of ad.

*Recognition.* As for recall, a significant main effect of ad content was found, *F*(2, 600) = 104.54, *p* < 0.001, with brand recognition highest for unhealthy food and lowest for healthy food (See Table 5 for means and Figure 8). Similarly, the ad *content* interacted with the *source* of the social media post, *F*(4, 600) = 32.67, *p* < 0.001 (Table 6 for pairwise comparisons and Figure 9 for interaction). For posts from celebrities and companies, unhealthy food brands were most frequently recognised, followed by non-food, with healthy food recognised least. As for recall, this effect was not observed for social media posts shared by peers: brand recognition of unhealthy and healthy food did not differ, and recognition for these was significantly higher than it was for non-food brands. There was no significant effect for age or internet use. Males remembered significantly more products than females in free recall, (*F*(1, 582) = 12.84; *p* < 0.001), but there were no gender differences in recognition.

## 4. Study 2: Eye-Tracking Measures of Attention

Study 1 had found that adolescents responded more positively to unhealthy food brands, compared to non-food brands and healthy food brands, in terms of social attitudes and memory. Study 2 measured adolescents’ attention to unhealthy food, healthy food and non-food advertising content, using eye-tracking. Dependent variables for Study 2 were (i) mean fixation duration and (ii) mean fixation count.

### 4.1. Participants and Procedure

Guided by power analysis and previous research sample sizes in eye-tracking studies, 81 participants (49 female) between the ages of 13 and 17 years (M = 15.37, SD = 1.351) were recruited for Study 2, through urban and rural Irish schools and youth groups between January and May 2017. All were Facebook users and 87.3% indicated they went online several times daily.

A Tobii-T60 eye-tracking monitor measured the number (count) and duration of fixations on the target posts. The stimuli were the same as for Study 1, with nine conditions (3 content × 3 source). Trial transitions were automated so participants did not need to scroll to proceed to the next stimulus. Each trial lasted 20 s with an opportunity to take a break every two minutes. After viewing the stimuli, participants were asked about their social media use.

### 4.2. Analyses and Results

#### 4.2.1. Analyses

First, the data were cleaned. Measures for participants with less than 80% usable eye-tracking data were reviewed by two researchers. Where data were missing for one of four trials per condition, participants’ scores for that trial were based on the average of the three available trial datum (eight occurrences across four participants). Where two or more of four trials were missing in one condition, that condition was counted as missing in the analysis (five occurrences across four participants). As a result, two participants were excluded entirely. One withdrew before completing the study, leaving 79 remaining participants. Next, the mean number and duration of participants’ fixations were coded and collated for each condition. Data were then analysed using general linear mixed model analyses. The variables of post content, post source, age, gender, and how often participants went online were included in the models as potential predictors.

#### 4.2.2. Advertisement Content: Unhealthy, Healthy, and Non-Food

**Figure 10:**
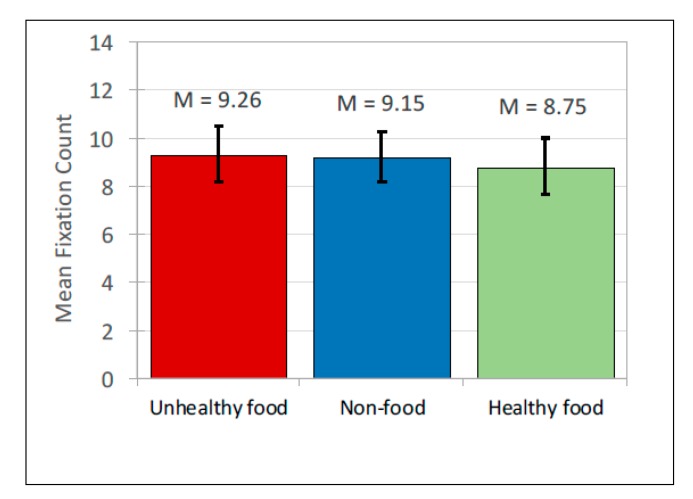
Fixation count: Mean scores.

**Figure 11:**
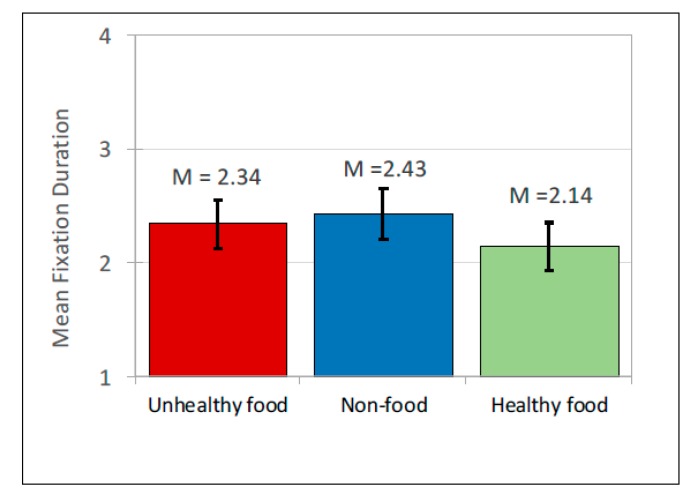
Fixation duration: Mean scores.

Advertisement post content had no significant main effect on fixation count, *F*(2, 167) = 0.282, *p* = 0.754 (Figure 10; Table 7 and Table 8). However advertisement post content had a significant main effect on fixation duration, *F*(2, 693) = 6.463, *p* = 0.002 and pairwise comparisons showed that healthy food items were attended to less than unhealthy food items, *t*(693) = −2.499, *b*(0.077) = −0.192, *p* = 0.013, and less than non-food items, *t*(693) = −3.404, *b*(0.083)= −0.284, *p* < 0.001 (Figure 11; Table 7 and Table 8). Fixation duration did not differ significantly between unhealthy and non-food items. Taken together, these findings indicate that participants looked at all types of advertising posts equally frequently, but looked at unhealthy and non-food advertisements for longer than they did at healthy food advertisements.

#### 4.2.3. Interactions between Content and Source of Advertisement

**Figure 12:**
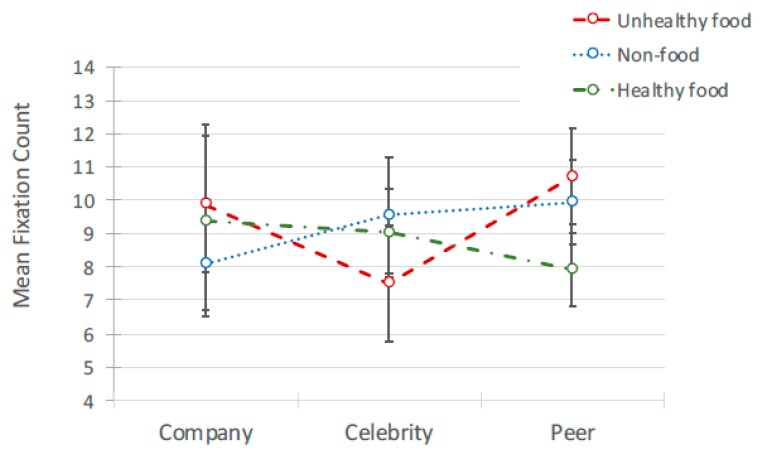
Interaction between fixation count and source of ad for 3 types of ad.

**Figure 13:**
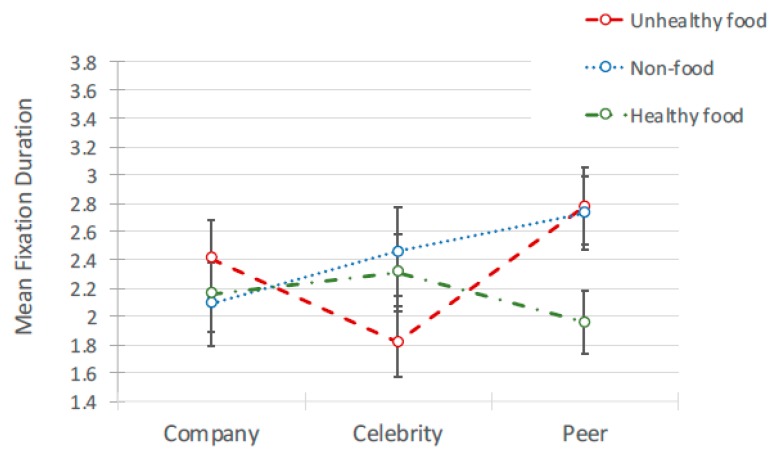
Interaction between fixation duration and source of ad for 3 types of ad.

There was a significant interaction between advertisement content (healthy, unhealthy non-food) and source (peer, celebrity, sponsored) for fixation duration *F*(4, 693) = 17.395, *p* < 0.001 and count *F*(4, 167) = 3.33, *p* = 0.012 (Figure 12 and Figure 13). Pairwise comparisons (Table 8) indicated that attention duration for advertisement content was different depending on whether the source was a peer or a celebrity. When posted by a peer, fixation durations were significantly longer for unhealthy food posts than for healthy food posts, *t*(693) = 6.773, *b*(0.121) = 0.817, *p* < 0.000, as were mean fixation counts, *t*(167) = 3.458, *b*(0.807) = 2.79, *p* < 0.000. However, the opposite pattern was found when posted by a celebrity, where fixation duration was significantly longer for healthy food ad posts than for unhealthy food ad posts, *t*(693)=3.652, *b*(0.134) = 0.491, *p* < 0.000.

#### 4.2.4. Age and Gender Effects

No main effects of gender were observed in the models for fixation duration, *F*(1, 693) = 0.968, *p* = 0.325, or for fixation count, *F*(1, 167) = 0.208, *p* = 0.649. However, age effects were observed for both: fixation duration, *F*(1, 693) = 4.010, *p* < 0.000, and fixation count, *F*(1, 167) = 5.934, *p* = 0.016, were greater for older adolescents, demonstrating that older adolescents paid more attention to the posts overall.

## 5. Discussion

This is the first study, as far as we are aware, to examine adolescent responses in social, memory, and attention domains to the same set of social media advertising for a range of products and sources. We found a consistent pattern. When young people stated which posts they *would share* in social media; when assessing their *attitude to peers*, (Study 1a); when attempting to *recall* brands they had seen; or *recognise* them from a list (Study 1b), the young people in this study responded significantly more positively to unhealthy food advertising compared to non-food advertising, and to both these product types significantly more positively than to healthy food advertising. Although free *recall* rates overall were low, recall for unhealthy food brands was nearly five times as great as for healthy food brands and nearly twice as great as for non-food brands. Furthermore, when prompted from a list that included distractors, young people *recognised* many unhealthy food brands and did so at approximately twice the rate of healthy food and non-food brands. Finally, for measures of attention (Study 2), adolescents did not differ in the types of ads they looked at (*fixation count*), but they looked at ads for unhealthy foods for significantly longer (*fixation duration*).

In addition to the multiple domains measured in this study, a further novel feature was that it examined interactions between the content of a social media advertising post and its source. Here the findings were less straightforward. The source of the advertising post did not affect young people’s likelihood to share it, nor did the source affect their attitudes to the fictional peer whose social media account they were viewing (Study 1a). This suggests that, for social responses to advertising content in social media, adolescents are as susceptible to effects when ads originate from a company or brand, as they are when ads are shared by celebrities or their peer group. However, when measuring memory for brands (Study 1b), and fixation duration (Study 2), source did interact with ad content, although the patterns here were contradictory for these two domains. The fixation duration findings suggest that young people attended to *unhealthy* food posts from their *peers* for longer than from other sources, and to *healthy* food advertising posts from *celebrities* for longer than from other sources. Yet when participants’ free recall for brands was measured, the findings were different. For ads shared by *celebrities* and *companies*, participants recalled significantly more unhealthy food brands than non-food or healthy food brands, yet for ads shared by *peers*, recall did not differ significantly. Similarly, for recognition, when ads were shared by *celebrities* and *companies*, participants recognised unhealthy food brands significantly more, followed by non-food, with healthy food recognised least; yet when ads were shared by *peers*, recognition of unhealthy and healthy food brands did not differ, and non-food brands were recognised significantly less than food brands.

Taken together, therefore, the study findings indicate that adolescents attended to all of the advertising they saw in social media, but they viewed unhealthy food advertising posts for longer. They also recalled unhealthy food brands more, recognised them more, were more likely to share them, and had more positive views of peers in whose social media accounts they saw unhealthy food advertising posts.

The findings regarding source of the post were complex and contradictory and the interactions between product type and the source of the advertising post warrant further investigation. As unhealthy food brands are frequently promoted by celebrities popular with adolescents [45,46], it is notable that we found that celebrity-shared posts for unhealthy brands were recalled significantly more frequently than posts from other sources. Interestingly, brand recall and recognition for unhealthy and healthy foods did not vary for peer-shared posts. This suggests the possibility that young adolescents may be most open to healthy food communications from their own peers, and therefore that if seeking to promote healthier practices in social media, peer-led social marketing [65] might be more likely to succeed than celebrity-led or public service messages.

Advertising recall and recognition is significant for children’s health as it is the first step in the hierarchy of food advertising effects that is hypothesised to lead to changes in eating behaviour and body weight gain [49]. Meta-analysis of 45 studies has concluded that viewing food images provokes as strong a desire to eat as exposure to food itself [66], and further analyses indicate that attentional bias towards food images is greater in those who eat more [57,67,68,69]. Furthermore, neural responses to fast food advertising predict intake [70].

The findings for attention were least straightforward of the three outcome domains in this study and this mirrors previous findings in this field [69,71].As eye-tracking measures eye movement behaviour as an indirect index of processing, it is difficult to separate out the relative contributions of expectation, pre-existing attitudes, and bottom-up perceptual features of the stimulus. Given the conditions included multiple trials with varied products and stimuli, we might infer that the present findings are driven by expectations about types of products and who posts them rather pre-existing knowledge of a product or perceptual features of a specific ad. At the same time, it could be argued that as food is an item children engage with daily from infancy, and as almost all existing advertising is for unhealthy foods, it is likely that in this domain, young people have already developed perceptual fluency for unhealthy food marketing which in turn increases liking [72] when presented with stimuli. Thus, even brief attention to unhealthy food advertisements may reinforce positive attitudes, and furthermore, less attention is required for recall for unhealthy, compared to healthy and non-food advertising [73]. The exact role and relationships of visual attention, perceptual fluency, existing implicit attitudes and subsequent behaviours remain to be clarified and offer rich potential for future research.

The findings for peer attitudes echo studies of young people’s views of unhealthy food in other contexts. For example, Swedish 14-year-olds, when creating their own images on Instagram, included significantly more images of unhealthy food on their online profiles than healthier options [40]; 13 to 15-year-olds at school in the UK believed healthy eating was ‘uncool’ and damaged the self-image they wanted to convey to peers [74]. As school friends’ Body Mass Indices (BMI) in Australian and American adolescents were related, with those of higher BMIs being most similar [75], participants’ greatest likelihood to share posts for unhealthy food is a concern as it indicates that unhealthy food marketing is the type young people are most likely to spread among networks.

Age was also pertinent to adolescents’ attention as older participants looked more and for longer at the advertising posts. The development of control systems as adolescents age allows them to retain attention for longer than younger adolescents [76]. They also spend more time viewing social media later in adolescence [7]. Although it is often posited that cognitive capacity to recognise advertising and its persuasive intent is fully developed by adolescence (see discussion in WHO, 2016 [6]), the findings of the present study indicate that attention paid to advertising posts increases with age, suggesting potential greater vulnerability.

Participants’ positive attitudes to peers with unhealthy food advertising content in social media, and their greater willingness to ‘share’ it, indicates that this form of advertising likely contributes to adolescent identity expression online [77]. Social identity theories describe how people come to develop a sense of self, or ‘who I am’, based on features of the social groups to which they belong; group identities are essential parts of the self-concept that “form a socially constructed sense of who and what ‘we’ are and also who and what ‘we’ are not” [78]. One of the ways in which adolescents establish an identity as distinct from older generations is through eating and identifying with widely marketed ‘junk’ foods, a form of adolescent identity expression that reflects marketing campaigns and that has been found in multiple cultures over decades [77]. As social media are sites in which adolescents engage in the central developmental task of identity formation [79], unhealthy food marketing is likely to become enmeshed in this process.

Adolescents’ attention to unhealthy food advertising, their recall of this advertising and their evaluation of peers and likelihood to share this content through their networks, all constitute important facets of their responses to advertising content in a media-saturated environment. These early findings for social media effects on young adolescents’ recall and recognition of food and other brands have implications for future research and policy action to protect and promote health. Before considering these, we address strengths and limitations of the study.

### 5.1. Limitations

Researchers examining digital media practices and effects face substantial design challenges. In contrast to broadcast media, users’ experiences in social media (including the advertising they see) are personalized, yet accessing young people’s actual social media accounts for research is particularly ethically challenging [6,80]. This study therefore featured fictional peers, and celebrities that are popular with participants’ age group. How this impacts on the ecological validity of the study is uncertain, although it seems reasonable to infer that effects might be greater for actual friends and celebrities they personally follow.

Furthermore, young people’s digital platforms preferences can change rapidly. Facebook was the most-used social media platform among Irish teens when the study was designed [8]; by the time it was carried out, Snapchat and Instagram had become increasingly popular [81], and at the time of writing, Snapchat had become less of a focus with the rise of TikTok [82]. Still, Facebook continues to be a widely used platform [7]. We are unaware of any studies comparing advertising effects across platforms. However, as the effects found in this study mirror those identified in television, it suggests that they are likely to transfer to other digital platforms as well.

### 5.2. Strengths

The study is the first we know of that examines young adolescents’ social responses, memory and attention of social media advertising posts for healthy, unhealthy and non-food brands in multiple social contexts. It benefits from integrating theory of social norms of food and social media with food marketing effects. The stimuli were designed with the input of young people, to closely simulate social media accounts. This feature, together with the fact that participants were blind to the aim of the study when viewing the feeds, was a major strength in the experiment, achieving a combination of ecological validity and levels of experimental control that are typically subjected to trade-off in research studies.

### 5.3. Implications for Research and Policy

This study adds to a nascent body of evidence indicating that food marketing in digital media is likely to contribute to adverse effects on adolescents’ health [83]. Of interest for research in advertising effects is the contrast between the free and prompted recall rates found. Reflecting findings for much younger children [84], this indicates that even when memory is more developed in adolescence, free recall remains a poor measure of advertising exposure compared to prompted recognition. Future areas of exploration are links between social responses to food marketing (sharing and peer assessment) and consumption patterns.

For policy, the findings indicate the likely vulnerability of young adolescents to food marketing. Engagement with food and beverage brands in social media is widespread: in the US, millions of adolescents follow these accounts [15] and 70% of 1564 adolescents surveyed reported engaging with at least one brand and 35% with five or more [85]. They will therefore receive food and beverage marketing, and the present study shows they are likely to share this with their networks. Furthermore, the impact of shared advertising for unhealthy products can be predicted to be disproportionately powerful, as adolescent social norms of eating and perception of others’ consumption skews towards unhealthy foods, and this in turn disproportionately affects adolescents’ eating [38].

Adolescents are under-represented in research regarding food marketing and are typically neglected by regulatory measures aimed at protecting children from the negative health effects of unhealthy food marketing [6]. This study indicates that the present global focus on protecting children up to 12 years old may leave a substantial proportion of young people, at the age when their social media use rises rapidly [7], unprotected from digital food marketing [86] and thus in a position where their rights to health, privacy and freedom from exploitation are infringed [87].

## 6. Conclusions

In conclusion, in social media, young people’s responses to unhealthy food advertising posts were significantly greater than their responses to unhealthy and non-food posts, whether measured by their attention, memory, positive assessment of peers, or likelihood to ‘share’. Given adolescents’ extensive use of social media, these findings are important in considering regulation of marketing beyond the age of 13 (currently the upper limit in many countries) and even beyond 15 (as in Ireland and the UK), as attention to advertising increased between 13 and 17 years. The study provides evidence that existing restrictions aiming to protect children from unhealthy food advertising in television should be extended to digital advertising seen by young adolescents in social media.

## Figures and Tables

**Table 1:** Celebrities that appeared as sources of advertising posts and number of followers on Facebook at the time of the study.

Females	Occupation	Followers	Males	Occupation	Followers
Rihanna	Barbadian Pop Star	82 m.	Cristiano Ronaldo	Portuguese Soccer Player	116 m.
Taylor Swift	US Pop star	75 m.	Justin Bieber	Canadian Pop Star	77 m.
Katy Perry	US Pop Star	71 m.	Will Smith	US Actor	75 m.
Adele	British Pop Star	66 m.	Dwayne Johnson	Canadian Actor	57 m.
Beyonce	US Pop Star	64 m.	Channing Tatum	US Actor	20 m.
Selena Gomez	US Pop Star	61 m.	Ed Sheeran	British Pop Star	15 m.
Emma Watson	British Actor	33 m.	Nick Jonas	US Pop Star	10 m.
Ariana Grande	US Pop Star	30 m.	Conor McGregor	Irish Boxing Star	4 m.
Jennifer Lawrence	US Actor	16 m.	James Corden	British TV Presenter	3 m.
Gal Gadot	Actor	8 m.	Chris Pratt	US Actor	3 m.
Jessica Alba	US Actor	5 m.	John Boyega	British Actor	0.3 m
Perrie Edwards	British Pop Star	0.7 m.	Pádraig Harrington	Irish Pro Golfer	0.05 m.

**Table 2:** Brands and products shown in target images.

Brand	Product
**Unhealthy Food**	
Ben & Jerry’s Ice Cream	Cinnamon Buns Ice Cream
Cadbury	Crème Egg
Kellogg’s	Coco Pops
Eddie Rockets	Burger and Chips
KFC	KFC Bucket Chicken
Subway	Sub Sandwich
McDonalds	BBQ Chicken and Bacon Wrap
Bunsen Restaurant	Burger and Chips
Supermac’s	Fresh Chicken Breast Meal
Apache Pizza	Large Pizza Deal
Doritos	Cool Breeze & Chilli Heatwave
Walkers	Potato chips
**Healthy Food**	
Keelings	Mixed Berries
Fyffes	Bananas
Chopped	Salad Bowls
Tesco	Swiss style muesli
Weetabix	Weetabix
Kellogg’s	Special K
Good4U	Super Seeds Snacks
Kelkin	Rice Cakes
Irish Pride	Healthy Grain
Uncle Bens	Wholegrain Rice
Dannon	Light and Fit Greek Yoghurt
John West	Tuna
**Non-Food**	
Himox	Bluetooth Speakers
Luckies	Smart Phone Projector
Sennheiser	Headphones
H&M	Clothes
Penneys	Jeans
Asos	T-Shirt
Adidas	Sneakers
Nike	Sneakers
Dior (f)	‘Pure Poison’ perfume
Honest Beauty (f)	Lip Crayons
Covergirl (f)	Mascara
Coco Brown (f)	Fake Tan
Under Armor (m)	Gym bag
PlayStation Uncharted4 (m)	Game
Nerf Gun (m)	Nerf Mastodon
Google (m)	Chromecast

**Table 3:** Attitude to peer and likelihood to share: Means, standard deviations, and 95% confidence intervals.

		Attitude to Peer			Likelihood to Share		
Content	Source	M	SD	95% CIs	M	SD	95% CIs
Unhealthy food	Peer	3.38	0.59	[3.24, 3.52]	2.53	0.903	[2.32, 2.74]
	Celebrity	3.44	0.50	[3.32, 3.56]	2.76	0.979	[2.53, 2.99]
	Company	3.27	0.57	[3.13, 3.40]	2.59	1.015	[2.35, 2.83]
	**All sources**	**3.36**	**0.48**	**[3.25, 3.47]**	**2.63**	**0.865**	**[2.42, 2.83]**
Healthy food	Peer	3.12	0.52	[3.00, 3.24]	1.91	0.728	[1.74, 2.08]
	Celebrity	3.26	0.61	[3.12, 3.40]	1.93	0.749	[1.76, 2.11]
	Company	3.17	0.51	[3.05, 3.29]	2.00	0.817	[1.81, 2.19]
	**All sources**	**3.18**	**0.47**	**[3.29, 0.47]**	**1.95**	**0.679**	**[1.79, 2.11]**
Non-food	Peer	3.34	0.58	[3.20, 3.47]	2.61	0.912	[2.40, 2.83]
	Celebrity	3.29	0.57	[3.16, 3.42]	2.52	0.918	[2.31, 2.74]
	Company	3.23	0.44	[3.12, 3.33]	2.47	0.950	[2.25, 2.69]
	**All sources**	**3.28**	**0.45**	**[3.39, 0.45]**	**2.54**	**0.798**	**[2.35, 2.72]**
All types	Peer	3.28	0.51	[3.16, 3.39]	2.35	0.721	[2.18, 2.52]
	Celebrity	3.33	0.48	[3.22, 3.44]	2.41	0.755	[2.23, 2.59]
	Company	3.22	0.43	[3.12, 3.32]	2.35	0.772	[2.17, 2.53]
	**All sources**	**3.274**	**0.055**	**[3.17, 3.382]**	**2.37**	**0.704**	**[2.17, 2.53]**

All sources: Mean of responses across all 3 source conditions.

**Table 4:** Attitude to peer and likelihood to share: Pairwise contrasts.

		Attitude to Peer		Likelihood to Share	
Group	Pairwise Contrasts	*t*(636)	*p*	*t*(636)	*p*
Unhealthy food					
	Peer vs Celebrity	−0.945	0.345	−2.321	0.021 *
	Celebrity vs Brand	2.849	0.005 *	1.595	0.111
	Brand vs Peer	−1.928	0.054 *	0.568	0.57
Healthy food					
	Peer vs Celebrity	−2.563	0.011 *	−0.255	0.799
	Celebrity vs Brand	1.503	0.133	−0.819	0.413
	Brand vs Peer	0.815	0.415	1.087	0.278
Non-food					
	Peer vs Celebrity	0.877	0.381	0.864	0.388
	Celebrity vs Brand	1.093	0.275	0.509	0.611
	Brand vs Peer	−1.873	0.062	−1.278	0.202
Peer					
	Unhealthy vs Healthy	4.951	<0.001 *	6.973	<0.001 *
	Healthy vs Non-food	−4.132	<0.001 *	−7.538	<0.001 *
	Non-food vs Unhealthy	−0.777	0.437	0.804	0.422
Celebrity					
	Unhealthy vs Healthy	2.941	0.003 *	8.931	<0.001 *
	Healthy vs Non-food	−0.484	0.628	−6.523	<0.001 *
	Non-food vs Unhealthy	−2.658	0.008 *	−2.365	<0.001 *
Brand					
	Unhealthy vs Healthy	1.627	0.104	6.118	<0.001 *
	Healthy vs Non-food	−0.949	0.343	−4.637	<0.001 *
	Non-food vs Unhealthy	−0.687	0.492	−1.053	0.293

* Significant at *p* < 0.05

**Table 5:** Recall and recognition: Means, standard deviations, and 95% confidence intervals for recalled and recognised brands.

		Recall			Recognition		
Content	Source	M	SD	95% CIs	M	SD	95% CIs
Unhealthy food	Peer	0.23	0.46	[0.12, 0.35]	2.28	1.08	[2.02, 2.54]
	Celebrity	0.67	0.71	[0.49, 0.85]	3.12	1.15	[2.84, 3.40]
	Company	0.84	0.84	[0.63. 1.05]	2.13	1.17	[1.85, 2.42]
	**All sources**	**1.75**	**1.35**	**[1.41, 2.09]**	**7.53**	**2.63**	**[6.89, 8.17]**
Healthy food	Peer	0.16	0.41	[0.05, 0.26]	2.18	1.20	[1.89, 2.47]
	Celebrity	0.05	0.23	[0, 0.10]	0.74	0.86	[0.53, 0.94]
	Company	0.16	0.37	[0.06, 0.25]	0.96	0.95	[0.73, 1.19]
	**All sources**	**0.36**	**0.60**	**[0.21, 0.51]**	**3.87**	**2.39**	**[3.29, 4.45]**
Non-food	Peer	0.28	0.55	[0.14, 0.42]	1.24	0.96	[1.00, 1.47]
	Celebrity	0.23	0.53	[0.10, 0.37]	1.56	1.10	[1.29, 1.82]
	Company	0.58	0.81	[0.38, 0.78]	1.65	1.12	[1.38, 1.92]
	**All sources**	**1.09**	**1.15**	**[0.81, 1.38]**	**4.44**	**2.48**	**[3.84, 5.04]**
All types	Peer	0.67	0.76	[0.48, 0.86]	5.69	2.35	[5.12, 6.26]
	Celebrity	0.95	0.90	[0.73, 1.18]	5.41	2.33	[4.85, 5.98]
	Company	1.58	1.28	[1.26, 1.90]	4.74	2.45	[4.14, 5.33]
	**All sources**	**3.20**	**1.90**	**[2.73, 3.68]**	**15.84**	**6.38**	**[14.30, 17.38]**

All sources: Mean of responses across all 3 source conditions.

**Table 6:** Recall and recognition: Results of pairwise contrasts.

		Recall		Recognition	
Group	Pairwise Contrasts	*t*(582)	*p*	*t*(600)	*p*
Unhealthy food					
	Peer vs Celebrity	−3.63	<0.001 *	−4.99	<0.001 *
	Celebrity vs. Brand	−1.18	0.239	5.74	<0.001 *
	Brand vs. Peer	4.66	<0.001 *	−0.89	0.377
Healthy food					
	Peer vs. Celebrity	2.06	0.04 *	8.76	<0.001 *
	Celebrity vs. Brand	−2.22	0.027 *	−1.7	0.091
	Brand vs. Peer	0.22	0.826	−7.43	<0.001 *
Non-food					
	Peer vs. Celebrity	0.49	0.623	−2.17	0.03 *
	Celebrity vs. Brand	−2.77	0.006 *	−0.59	0.558
	Brand vs. Peer	2.38	0.018 *	2.7	0.007 *
Peer					
	Unhealthy vs. Healthy	1.32	0.189	0.62	0.534
	Healthy vs. Non-food	−1.74	0.083	5.7	<0.001 *
	Non-food vs. Unhealthy	0.53	0.595	−6.3	<0.001 *
Celebrity					
	Unhealthy vs. Healthy	6.18	<0.001 *	12.62	<0.001 *
	Healthy vs. Non−food	−2.66	0.008 *	−5.78	<0.001 *
	Non- food vs. Unhealthy	−3.43	0.001 *	−8.98	<0.001 *
Brand					
	Unhealthy vs. Healthy	5.59	<0.001 *	7.17	<0.001 *
	Healthy vs. Non-food	−3.71	<0.001 *	−4.64	<0.001 *
	Non-food vs. Unhealthy	−1.34	0.18	−2.98	0.003 *

* Significant at *p* < 0.05

**Table 7:** Fixation count and duration: Means, standard deviations, and 95% confidence intervals.

		Fixation Duration			Fixation Count		
Content	Source	M	SD	95% CIs	M	SD	95% CIs
Unhealthy food	Peer	2.78	1.23	[2.51, 3.05]	10.71	6.09	[9.371, 12.234]
	Celebrity	1.82	1.13	[1.57, 2.07]	7.50	7.33	[5.963, 9.427]
	Company	2.41	1.24	[2.13, 2.68]	9.88	8.68	[8.038, 12.132]
	**All sources**	**2.34**	**0.969**	**[2.12, 2.55]**	**9.26**	**4.97**	**[8.161, 10.495]**
Healthy food	Peer	1.96	1.01	[1.74, 2.19]	7.92	4.73	[6.883, 9.106]
	Celebrity	2.31	1.24	[2.04, 2.59]	9.02	5.59	[7.801, 10.431]
	Company	2.16	1.21	[1.89, 2.43]	9.38	12.09	[6.933, 12.685]
	**All sources**	**2.14**	**0.951**	**[1.93, 2.35]**	**8.75**	**4.99**	**[7.656, 9.998]**
Non-food	Peer	2.73	1.18	[2.47, 2.99]	9.93	5.40	[8.743, 11.282]
	Celebrity	2.46	1.40	[2.15, 2.77]	9.55	7.43	[7.956, 11.456]
	Company	2.09	1.32	[1.80, 2.39]	8.09	5.91	[6.816, 9.598]
	**All sources**	**2.43**	**1.01**	**[2.20, 2.65]**	**9.15**	**4.38**	**[8.183, 10.24]**
All types	Peer	2.49	0.951	[2.28, 2.70]	9.44	3.83	[8.585, 10.386]
	Celebrity	2.20	1.00	[1.98, 2.42]	8.64	4.49	[7.652, 9.763]
	Company	2.22	1.00	[2.00, 2.44]	9.08	5.67	[7.846, 10.512]
	**All sources**	**2.30**	**0.886**	**[2.11, 2.50]**	**9.05**	**3.52**	**[8.261, 9.914]**

All sources: Mean of responses across all 3 source conditions.

**Table 8:** Fixation count and duration: Results of pairwise contrasts.

		Duration		Count	
Group	Pairwise Contrasts	*t*(693)	*p*	*t*(167)	*p*
Unhealthy food					
	Peer vs. Celebrity	7.233	<0.001 *	3.069	<0.001 *
	Celebrity vs. Brand	−4.38	<0.001 *	−1.845	0.067
	Brand vs. Peer	−2.559	0.011 *	−0.747	0.456
Healthy food					
	Peer vs. Celebrity	−2.839	0.005 *	−1.451	0.149
	Celebrity vs. Brand	1.061	0.289	−0.239	0.812
	Brand vs. Peer	1.672	0.095	0.976	0.33
Non-food					
	Peer vs. Celebrity	1.712	0.087	0.396	0.692
	Celebrity vs. Brand	2.147	0.032 *	1.418	0.158
	Brand vs. Peer	−4.273	<0.001 *	−2.337	0.021 *
Peer					
	Unhealthy vs. Healthy	6.773	<0.001 *	3.458	<0.001 *
	Healthy vs. Non-food	−6.703	<0.001 *	−2.752	0.007 *
	Non-food vs. Unhealthy	−0.369	0.712	−0.954	0.341
Celebrity					
	Unhealthy vs. Healthy	−3.652	<0.001 *	−1.477	0.142
	Healthy vs. Non-food	−0.915	0.36	−0.526	0.6
	Non-food vs. Unhealthy	4.212	<0.001 *	1.756	0.081
Brand					
	Unhealthy vs. Healthy	1.746	0.081	0.291	0.771
	Healthy vs. Non-food	0.43	0.667	0.837	0.404
	Non-food vs. Unhealthy	−2.029	0.043 *	−1.557	0.121

* Significant at *p* < 0.05.
